# Improved Efficacy of Transcatheter Arterial Chemoembolization Using Warmed Miriplatin for Hepatocellular Carcinoma

**DOI:** 10.1155/2014/359296

**Published:** 2014-09-08

**Authors:** Daisuke Yasui, Satoru Murata, Shiro Onozawa, Takahiko Mine, Tatsuo Ueda, Fumie Sugihara, Chiaki Kawamoto, Eiji Uchida, Shin-ichiro Kumita

**Affiliations:** ^1^Department of Radiology/Center for Advanced Medical Technology, Nippon Medical School, 1-1-5 Sendagi, Bunkyo-ku, Tokyo 113-8603, Japan; ^2^Division of Gastroenterology and Hepatology, Department of Internal Medicine, Nippon Medical School, 1-1-5 Sendagi, Bunkyo-ku, Tokyo 113-8603, Japan; ^3^Department of Surgery, Nippon Medical School, 1-1-5 Sendagi, Bunkyo-ku, Tokyo 113-8603, Japan

## Abstract

The aim of this study was to evaluate the efficacy and safety of transcatheter arterial chemoembolization (TACE) using warmed and nonwarmed miriplatin for hepatocellular carcinoma. Eighty patients (117 nodules), treated between January 2010 and June 2013, were evaluated. Thirty-two and 85 nodules were treated with nonwarmed and warmed miriplatin, respectively. The efficacy of TACE was evaluated on a per nodule basis according to treatment effect (TE). Adverse events were evaluated according to the Common Terminology Criteria for Adverse Events (CTCAE) v4.0. TE grades were significantly improved in the warmed group compared to the nonwarmed group (nonwarmed: TE 4, 12.5%; TE 3, 0%; TE 2, 15.6%; TE 1, 71.9%; warmed: TE 4, 34.1%; TE 3, 5.9%; TE 2, 9.4%; TE 1, 50.6%; *P* = 0.017) . Multivariate analysis revealed significant impact of warming miriplatin on objective response rate (odds ratio, 12.35; 95% confidence interval, 2.90–90.0; *P* = 0.0028). CTCAE grades of elevated aspartate and alanine transaminase after TACE were significantly higher in the warmed group (*P* = 0.0083 and 0.0068, resp.); however, all adverse events were only transient. The use of warmed miriplatin in TACE significantly improved TE without causing serious complications.

## 1. Introduction

Transcatheter arterial chemoembolization (TACE) is a standard therapy for intermediate stage unresectable hepatocellular carcinoma (HCC) [1]. Previous randomized controlled studies have shown that TACE prolongs survival and controls symptoms of HCC [2, 3].

Doxorubicin, epirubicin, cisplatin, and mitomycin C have been widely used as chemotherapeutic agents, either alone or in combination [4]. It is known that epirubicin, cisplatin, and mitomycin C can cause arteritis after injection, leading to hepatic artery occlusion and development of extrahepatic collateral pathways [5]. This change in vascular anatomy can make repetitive TACE difficult and limits the long-term efficacy of TACE.

A new platinum agent, miriplatin ((*SP*-4-2)-[(1*R*,2*R*)-cyclohexane-1,2-diamine-*N,N*′] bis (tetradecanoato-*O*) platinum monohydrate; Dainippon Sumitomo Pharma, Osaka, Japan), was recently developed [6, 7]. It is a lipophilic platinum complex that can be easily suspended in Lipiodol (Guerbet, Aulnay-sous-Bois, France) and gradually releases active platinum compounds in tumor tissue [7]. According to the experience in our facility (296 sessions of TACE using miriplatin in the past 4 years), miriplatin is likely to cause minimal damage to the hepatic artery (unpublished data). Thus, TACE using miriplatin can be performed repeatedly as needed [8].

Another advantage of miriplatin is its less severe toxicity profile compared to other agents, resulting from gradual release of platinum into serum [9, 10].

Less damage to feeding arteries and less severe adverse effects make miriplatin suitable for TACE; however, when compared to other agents, the clinical outcomes of TACE using miriplatin have not been satisfactory [11–13]. It has been reported that the high viscosity and the large oil droplet of miriplatin can cause early occlusion of feeding vessels, leading to inadequate accumulation in tumors [11]. The viscosity of miriplatin suspension has been shown to decrease as temperature increases, dropping from 50 mPa*·*s at 25°C to 12 mPa*·*s at 60°C [14, 15]. Therefore, local tumor control could be improved by using warmed miriplatin in TACE, as warming miriplatin will reduce its viscosity and increase miriplatin accumulation in the tumor.

Thus, the purpose of this study was to evaluate and compare the efficacy of nonwarmed miriplatin versus warmed miriplatin in TACE and to review the adverse events in both treatment groups.

## 2. Materials and Methods

This study was approved by the local institutional review board. The review board waived the need for informed consent given the retrospective design of the study.

### 2.1. Patients

**Figure 1:**
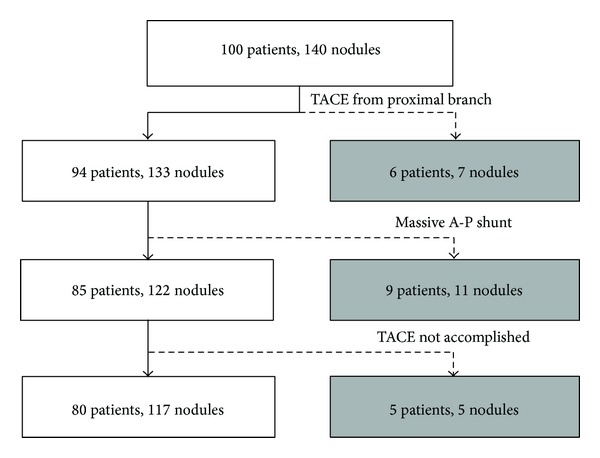
Patient enrollment. Gray boxes indicate patients excluded from the study. TACE: transcatheter arterial chemoembolization; A-P: arterioportal.

Patients were eligible for this retrospective study if they were diagnosed with HCC by either contrast-enhanced dynamic computed tomography or dynamic magnetic resonance imaging using gadolinium ethoxybenzyl diethylenetriamine pentaacetic acid. A total of 100 patients (140 nodules) were initially selected and met all the following requirements: 1 nodule per hepatic segment and a well-demarcated and hypervascular lesion (Figure 1). Among those, 20 patients (23 nodules) were excluded for the following reasons: 6 patients (7 nodules) for difficulty in selective catheterization of the feeding artery (beyond the second branch of the proper hepatic artery); 9 patients (11 nodules) for massive arterioportal shunts (A-P shunt) in which a segmental portal vein was visualized on hepatic arteriography; and 5 patients (5 nodules) for inadequate miriplatin accumulation in the target lesion because of large tumor size or anastomosis with vital vessels (pericardial and pulmonary veins) (Figure 1).

A total of 80 patients (117 nodules) were finally selected (Figure 1). TACE with nonwarmed miriplatin was performed on 22 patients (32 nodules) between January 2010 and December 2010. This included 17 men and 5 women, with a median age of 68 years (range, 51–83 years) (Table 1). TACE using warmed miriplatin was initiated in January 2011 in order to improve local tumor control by reducing viscosity. Fifty-eight patients (85 nodules) were treated with warmed miriplatin between January 2011 and June 2013. This included 34 male and 24 female patients, with a median age of 73 years (range, 50–91 years) (Table 1).

### 2.2. Treatment Procedure

The entire treatment procedure was performed under local anesthesia by administering lidocaine subcutaneously. A 4-French sheath (Super Sheath; Medikit, Miyazaki, Japan) was inserted via the femoral artery. Feeding arteries were routinely selected beyond the second branch of the proper hepatic artery and were cannulated with 2.0-French microcatheters (Gold Crest-MRT; Koshin medical, Tokyo, Japan). The miriplatin suspension was prepared by directly mixing miriplatin powder with lipiodol. The miriplatin/lipiodol suspension was prepared at 25°C for the nonwarmed miriplatin group. For preparing warmed miriplatin, lipiodol was mixed with miriplatin powder first and then the miriplatin/lipiodol suspension was immersed in a hot water bath for more than 5 minutes, which was kept at 55°C as measured using a thermometer inside a clean container placed in an electric range. The stability of miriplatin/lipiodol suspension at this temperature was confirmed by the manufacturer. The standard full dosage of the TACE protocol was miriplatin 120 mg. The miriplatin/lipiodol suspension was administered slowly under fluoroscopic guidance immediately after preparation without causing reflux, until the vascular bed of the target nodule was fully filled with the suspension, as confirmed under fluoroscopy. Thus, the amount of the miriplatin/lipiodol suspension was not predetermined, but rather decided by angiographic findings. Finally, the feeding arteries were embolized with ready-made 2 mm pieces of gelatin sponge (Gelpart; Nippon Kayaku, Tokyo, Japan), until complete stasis of the feeding arteries was obtained.

### 2.3. Evaluation of Treatment Effect

The response to TACE was evaluated on a per nodule basis, according to the 4-grade system: treatment effect (TE) grades 1–4 [16]. Pretreatment nodule size was measured using the most recent image (within 3 months) prior to the first treatment. The product of long and short axes length in the maximum cross section was calculated for each nodule before and after TACE, and nodules were classified according to the change in size: grade 4 (TE 4), 100% reduction in size or complete tumor necrosis; grade 3 (TE 3), 50% to 100% reduction in size; grade 2 (TE 2), <50% reduction to <25% increase in size; and grade 1 (TE 1), more than 25% increase in size. Objective response rate (ORR), defined as the proportion of TE 3 and 4 cases to the total, and disease control rate (DCR), defined as the proportion of TE 2, 3, and 4 cases to the total, were compared between the nonwarmed and warmed miriplatin groups. The dose of miriplatin used in each session was also compared between the 2 groups. Multivariate analysis was performed to identify factors that had significant influence on ORR.

### 2.4. Evaluation of the Effect of Anticancer Agents on the Hepatic Artery

Arterial damage to the hepatic artery was defined as vessel irregularity, stenosis, or occlusion. It was evaluated on a per regimen basis, independent of other analyses. All patients were assigned to three groups according to anticancer agent used in TACE: epirubicin/cisplatin, nonwarmed miriplatin, and warmed miriplatin, considering both past treatment and the treatment included in this study. Therefore, patients with multiple treatment history using different agents were assigned to more than one group. Preoperative arterial damage was evaluated with celiac arteriography prior to the treatment using each agent. Postoperative celiac arteriography before the usage of other agents was compared with the preoperative one. Damage to the hepatic artery was evaluated according to the 5-grade system: grade 0, no obvious damage; grade 1, irregular vessel wall; grade 2, vessel narrowing; grade 3, stenosis; and grade 4, occlusion. Severe damage was defined as grade 3 and grade 4. The level of arterial damage was classified into 4 levels: level 1, the proper hepatic artery; level 2, lobar branches; level 3, segmental branches; and level 4, subsegmental branches. Development of A-P shunt was also evaluated. The evaluation was performed by two observers (D.Y and T.M), independently and blinded to each other. After individual evaluation, the findings were disclosed and discrepancy in the findings was discussed by 2 observers.

### 2.5. Follow-Up

Either contrast-enhanced computed tomography or magnetic resonance imaging was performed every 3 to 6 months after TACE. The end of the follow-up period was defined as either the last patient visit or the addition of other treatments: TACE with other agents, radiofrequency ablation, or surgery. The entire follow-up period was completed in September 2013.

### 2.6. Adverse Events

Common Terminology Criteria for Adverse Events (CTCAE) version 4.0 was used to evaluate the safety of TACE using warmed miriplatin. Adverse events were evaluated on a per treatment session basis. Levels of aspartate transaminase (AST), alanine transaminase (ALT), total bilirubin, and complete blood counts were measured, and pre- and postoperative values were compared between the 2 groups. Eosinophilia, which is among the characteristic adverse events of miriplatin (defined as more than 450 cells/*μ*L), was also evaluated. Data obtained closest to the first treatment (within 1 month) were used as preoperative values. Either peak or trough data after the procedure were evaluated. Abnormal values were monitored to check for their return to the baseline. Incidence of constitutional symptoms, such as pyrexia and vomiting, was reviewed. Severe complications, such as liver failure, liver infarction, liver abscess, and bile duct necrosis, were also reviewed.

### 2.7. Statistical Analysis

Statistical analysis was performed using R 2.15.1 (CRAN: the Comprehensive R Archive Network at http://cran.r-project.org/). The parameters related to the patient and nodule characteristics, details of treatment, and adverse events were assessed with the Student's *t*-test, the Mann-Whitney *U*-test, and the Fisher's exact test. TE grades were assessed with the Mann-Whitney *U*-test. Logistic regression analysis was performed to identify factors that had significant impact on ORR, among potential prognostic factors: sex, hepatitis B virus infection, tumor size, values of *α*-fetoprotein (AFP) and des-gamma-carboxyprothrombin (DCP), Barcelona Clinic Liver Cancer (BCLC) stage, history of TACE, warming miriplatin, dose of miriplatin, and preoperative severe hepatic arterial damage [17–19]. Two-way ANOVA test was performed to reveal interaction between previous treatment history or preoperative severe arterial damage and warming miriplatin. Kappa value was calculated to assess the degree of interobserver agreement in evaluation of the hepatic arterial damage.

## 3. Results

### 3.1. Demographic Data and Tumor Profiles

Demographic data and parameters related to the patients and nodules are summarized in Table 1. No significant differences in age, sex, etiology of underlying chronic liver disease, Child-Pugh score, BCLC stage, or performance status (Eastern Cooperative Oncology Group classification) were observed between the nonwarmed and warmed miriplatin groups (*P* = 0.086, 0.10, 0.093, 0.73, 0.72, and 0.15, resp.). Preoperative AFP and DCP values were not available in 9 cases. No significant difference was observed in AFP and DCP values and nodule size (the product of long and short axes length) (*P* = 0.80, 0.15, and 0.72, resp.).

Eighteen cases (81.8%) and 32 cases (55.2%) had a previous history of TACE in the nonwarmed and warmed miriplatin groups, respectively (Table 1). Conventional lipiodol-TACE was performed using epirubicin, cisplatin, or miriplatin in previous sessions. Gelatin sponge was used as embolization material. The ratio of nodules with previous TACE history was significantly higher in the nonwarmed miriplatin group (*P* = 0.038). No significant difference was observed in the number of previous treatment sessions between the 2 groups (*P* = 0.087).

No significant difference was observed in follow-up period (*P* = 0.68). More than 80% of the nodules were followed up for more than 3 months and about half of the nodules were followed up for more than 6 months: longer follow-up period than previous studies [20, 21]. Frequency of preoperative severe arterial damage was significantly higher in the nonwarmed group (*P* = 0.037). There was no significant difference in the interval between preoperative image evaluation and treatment between the 2 groups (*P* = 0.084).

### 3.2. Treatment Effect

**Figure 2:**
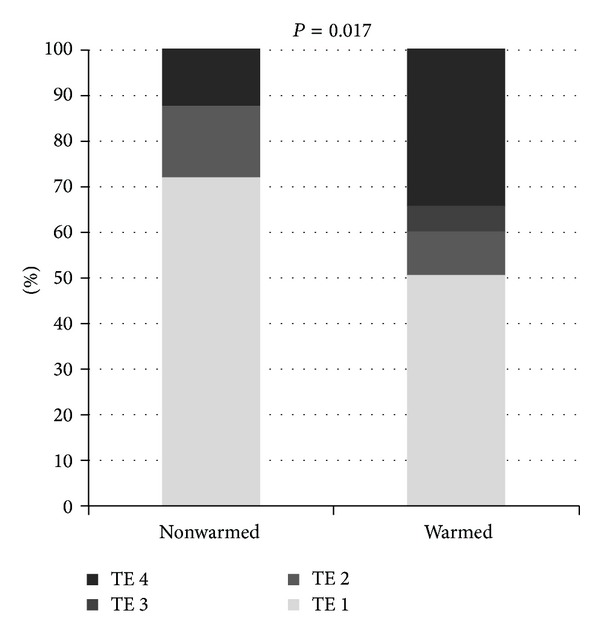
Treatment effect. The bar graph shows the distribution of treatment effect (TE) grades.

**Figure 3:**
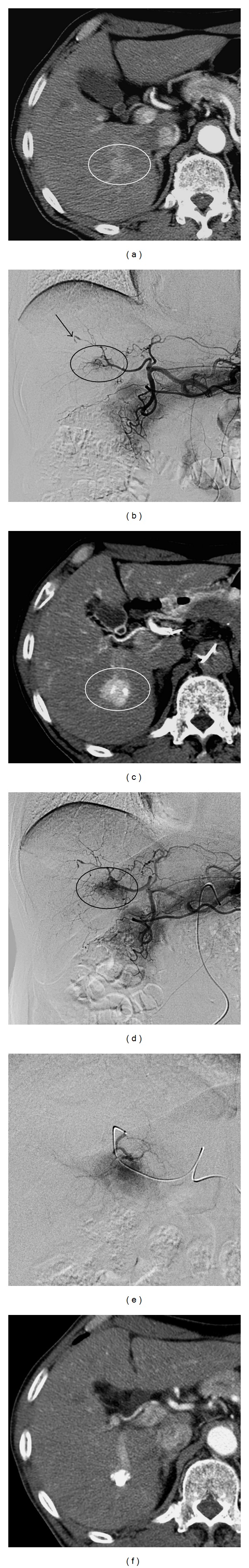
A case of hepatocellular carcinoma treated with warmed miriplatin. (a) Arterial phase of contrast-enhanced computed tomography (CT) before treatment. The white circle shows an enhanced lesion compatible with hepatocellular carcinoma. (b) Common hepatic arteriography showing a tumor stain (black circle). Occlusion and aneurysm formation are noted in the hepatic arterial branch, presumably caused by previous transcatheter arterial chemoembolization (TACE) using cisplatin (black arrow). (c) Early phase of CT during arteriography with the catheter tip placed in the common hepatic artery, 6 months after the first session of TACE using nonwarmed miriplatin. Obvious enhancement was observed, which indicated recurrence (white circle). (d) Celiac arteriography showing tumor stain (black circle). (e) Selective angiography with a microcatheter placed in a feeding artery. TACE was performed using warmed miriplatin in this session. (f) Arterial phase of contrast-enhanced CT, 4 months after the second session of TACE. Lipiodol accumulated densely in the target lesion, and the tumor size was decreased with no evidence of recurrence.

Warming miriplatin had an impact on TE grades. TE grades were significantly higher in the warmed miriplatin group than in the nonwarmed miriplatin group (*P* = 0.017; Figure 2). In the nonwarmed miriplatin group, 4 lesions were classified as TE 4 (12.5%), 0 as TE 3 (0%), 5 as TE 2 (15.6%), and 23 as TE 1 (71.9%); thus, ORR and DCR were 12.5% and 28.1%, respectively. In contrast, in the warmed miriplatin group, 29 lesions were classified as TE 4 (34.1%), 5 as TE 3 (5.9%), 8 as TE 2 (9.4%), and 43 as TE 1 (50.6%); thus, ORR and DCR were 40.0% and 49.4%, respectively. ORR was significantly higher in the warmed group (*P* = 0.0042), while there was no significant difference in DCR (*P* = 0.059). TACE using both nonwarmed miriplatin and warmed miriplatin was performed on 3 nodules. TE 1 was obtained in all cases after TACE using nonwarmed miriplatin. Treatment effect improved to TE 4 in 1 case (Figure 3); however, no improvement was observed in the other 2 nodules after TACE using warmed miriplatin. No significant difference was observed in the amount of administered miriplatin (33.5 ± 16.7 mg in the nonwarmed miriplatin group versus 42.9 ± 29.8 mg in the warmed miriplatin group; *P* = 0.18).

Logistic regression analysis revealed that warming miriplatin had a significant impact on ORR (odds ratio, 12.35; 95% confidence interval, 2.90–90.0; *P* = 0.0028; Table 2). Other factors did not have a significant impact on ORR.

Since significant difference was observed in previous treatment history and preoperative severe arterial damage, two-way ANOVA test was performed to reveal interaction between these factors and warming miriplatin. No significant interaction was observed between previous treatment history and warming miriplatin (*P* = 0.24) and between preoperative severe hepatic arterial damage and warming miriplatin (*P* = 0.38).

### 3.3. Angiographic Evaluation after TACE

Forty-three cases were included in the epirubicin/cisplatin treatment group. Thirty cases were included in the nonwarmed miriplatin group, considering 8 cases in the warmed miriplatin group with past treatment history using nonwarmed miriplatin. Postoperative angiography was not available in 12 cases in the warmed miriplatin group; therefore, 46 cases were included.

Discrepancy in arterial damage grade evaluation was observed in 7 cases, while that in arterial damage level evaluation was observed in 2 cases with the epirubicin/cisplatin group (*κ* value: 0.71 and 0.77, resp.). Discrepancy in arterial damage grade evaluation was observed in 2 cases with both nonwarmed and warmed miriplatin groups (*κ* value: 0.85 and 0.70, resp.). Agreement on arterial damage level was obtained in all cases with the nonwarmed and warmed miriplatin groups. Agreement on A-P shunt formation was obtained in all cases with all groups. Discrepancy in the findings was discussed by two observers and consensus was formed in all cases.

According to the consensus, severe arterial damage was observed in 12 of 43 cases (27.9%) with the epirubicin/cisplatin group, in 2 of 30 cases (6.7%) with the nonwarmed miriplatin group, and in 0 of 46 cases (0%) with the warmed miriplatin group (Table 3). There was no significant difference in the number of treatment sessions (*P* = 0.164).

### 3.4. Adverse Events

Severe complications, such as liver abscess, bile duct necrosis, and liver infarction, and complications above CTCAE grade 4 were not observed in either group. There was no 30-day mortality.

Grades of AST and ALT elevation after treatment were significantly higher in the warmed miriplatin group (*P* = 0.0083 and 0.0068, resp.; Table 4), although no significant difference was observed in preoperative values (*P* = 0.14 and 0.32, resp.). Return to the preoperative level within 1 month was observed in all cases, with the exception of 1 and 2 cases in the nonwarmed and warmed miriplatin groups, respectively, in which sustained mild elevation of AST and ALT (80–150 IU/L) was observed. These cases were managed conservatively.

No significant difference was observed in grades of anemia (*P* = 0.060; Table 4); however, transfusion was necessary in 2 cases. No obvious relationship between anemia and TACE was identified, and recovery to the preoperative level was observed in all cases within 1 month. Data on eosinophilia was missing in 2 sessions with the nonwarmed miriplatin group and in 25 sessions with the warmed miriplatin group. No significant difference was observed in occurrence of eosinophilia (*P* = 0.26). No significant difference was observed in other parameters.

## 4. Discussion

In this study, the improved efficacy of warmed miriplatin compared to nonwarmed miriplatin when used in TACE for HCC was demonstrated; both TE and ORR were improved in the warmed miriplatin group. DCR was also better in the warmed miriplatin group, although this difference was not significant. No significant difference was observed in patient profiles or parameters related to each nodule, except history of TACE and preoperative severe hepatic arterial damage, which was more frequent in the nonwarmed miriplatin group. However, logistic regression analysis revealed that these parameters had no significant impact on objective response. Two-way ANOVA tests also revealed that there was no significant interaction between these parameters and warming miriplatin. On the contrary, Seko et al. reported that history of TACE had significant impact on tumor response [20]. This discrepancy in conclusion can be attributed to the difference in evaluation method (modified RECIST versus TE), since tumor response can be evaluated differently with different criteria. Furthermore, they did not consider hepatic arterial damage, which can have direct impact on tumor response. Therefore this study gives more comprehensive analysis; however, prospective trial with matched background is desirable to obtain appropriate conclusion.

TACE-induced hepatic arterial damage is a factor that limits the efficacy of this treatment. Good interobserver agreement was obtained in evaluation of the hepatic arterial damage grade and level. Severe arterial damage, which can interfere with injection of anticancer agents and lipiodol, was less frequent with nonwarmed and warmed miriplatin, compared to epirubicin/cisplatin (6.7% and 0% versus 27.9%). More proximal arterial damage was observed with epirubicin/cisplatin. These facts suggest that miriplatin is more suitable for TACE due to its less severe arterial insult.

However, previous studies have shown inferior local tumor control of TACE using miriplatin compared to other agents, including cisplatin and epirubicin [11–13]. Iwazawa et al. reported that the high viscosity of miriplatin suspension may result in early occlusion of tumor feeders before sufficient accumulation of miriplatin in the tumor is obtained, and this may be a major factor related to inferior local tumor control [11]. The viscosity of miriplatin has been shown to decrease with increasing temperature [14, 15]; a few studies have revealed improved local tumor control when investigating the efficacy of TACE using warmed miriplatin [20, 21]. However, these studies showed only the short-term TE (less than 3 months); in contrast, our study investigated the longer-term TE (8.5 and 7.7 months in average with the nonwarmed and warmed miriplatin groups, resp.). Another advantage of our study is its practical study design; nodules were followed up until treatments other than miriplatin-TACE were performed. Thus, the efficacy of miriplatin-TACE was genuinely evaluated.

The reason why local tumor control can be improved when using warmed miriplatin remains unclear. According to the hypothesis that agents with high viscosity cause proximal occlusion of feeding arteries, warmed miriplatin, which is therefore less viscous, can be injected to more distal parts of feeding arteries. Distal vessels usually have more vascular beds than proximal vessels, and thus the amount of injected miriplatin suspension may be assumed to increase; however, there was no significant difference in the miriplatin dose between the nonwarmed and warmed miriplatin groups in our study. Additional studies should be performed to reveal the mechanism underlying the improved efficacy of TACE using warmed miriplatin.

Despite the promising results, local tumor control in this study was not comparable to that found in previous studies on TACE using miriplatin [7, 9–12]. This may be attributed to the profile of cases included in this study. The levels of tumor markers investigated in our study (AFP and DCP) were quite high, and these markers are known to be negative prognostic factors given their association with aggressive pathological features [22–24]. Previous clinical studies have also shown that cases with high AFP levels have poor prognoses [25, 26].

With regard to adverse events, CTCAE grades of AST and ALT elevation were significantly higher in the warmed group. There is no consensus on whether elevation of transaminase levels after treatment is caused by damage to normal liver parenchyma or tumor necrosis, so it is difficult to interpret these data. Elevation of transaminase levels was only transient and was managed conservatively. No significant difference was observed in other parameters. Moreover, no serious complications, such as liver infarction, liver abscess, and bile duct necrosis, were observed in either group. These findings indicate that adverse events of TACE using warmed miriplatin are only transient.

Our findings provide evidence that warmed miriplatin can improve the efficacy of TACE; however, this study has some limitations. First, it is a retrospective study, and a randomized controlled study should be considered to further support these findings. There was asymmetry in the numbers of nodules treated in the nonwarmed and warmed groups, which was inevitable given the nature of a retrospective study. This study presented preliminary results on the efficacy of TACE using warmed miriplatin, and a prospective clinical trial is going to be conducted. Second, in order to establish the advantage of using miriplatin, the relationship between survival benefit and repeatability of TACE should be demonstrated. Moreover, the efficacy of TACE using warmed miriplatin should be compared with TACE using other agents or microspheres.

## 5. Conclusions 

This study demonstrated the safety and improved efficacy of TACE using warmed miriplatin compared to nonwarmed miriplatin for the treatment of HCC.

## Figures and Tables

**Table 1:** Profile of patients and nodules.

	Nonwarmed group (*n* = 22)	Warmed group (*n* = 58)	*P* value
Age, years	68 (51–83)	73 (50–91)	0.086
Sex, *n* (%)			
Male/female	17/5 (77.3/22.7%)	34/24 (58.6/41.4%)	0.10
Etiology, *n* (%)			0.093
HCV	14 (63.6%)	44 (75.9%)	
HBV	1 (4.5%)	7 (12.1%)	
Alcohol	5 (22.7%)	3 (5.2%)	
Others	2 (9.2%)	4 (6.8%)	
Child-Pugh, *n* (%)			0.73
5-6 (Class A)	13 (59.1%)	33 (56.9%)	
7–9 (Class B)	8 (36.4%)	23 (39.7%)	
10 (Class C)	1 (4.5%)	2 (3.4%)	
Median score	6.4	6.5	
BCLC stage, *n* (%)			0.72
0 (very early)	0 (0%)	0 (0%)	
A (early)	0 (0%)	0 (0%)	
B (intermediate)	8 (36.4%)	21 (36.2%)	
C (advanced)	12 (54.5%)	36 (62.1%)	
D (terminal)	2 (9.1%)	1 (1.7%)	
PS, *n* (%)			0.15
0	6 (27.3%)	23 (39.7%)	
1	13 (59.1%)	33 (56.9%)	
2	2 (9.1%)	2 (3.4%)	
3	1 (4.5%)	0 (0%)	
AFP (ng/mL)	282.8 ± 723.1	415.5 ± 1454.3	0.80
DCP (mAU/mL)	1562.6 ± 5241.2	581.3 ± 2913.8	0.15
Nodule size (mm^2^)	388.6 ± 398.0	383.1 ± 468.0	0.72
History of TACE	18 (81.8%)	32 (55.2%)	0.038∗
Agent			
EPI alone	8/18 (44.4%)	4/32 (12.5%)	
CDDP alone	6/18 (33.3%)	12/32 (37.5%)	
MPT alone	0/18 (0%)	5/32 (15.6%)	
Multiple agents	4/18 (22.3%)	11/32 (34.4%)	
Number of sessions			0.087
1	11/18 (61.1%)	17/32 (53.1%)	
2	4/18 (22.2%)	9/32 (28.1%)	
3	1/18 (5.6%)	4/32 (12.5%)	
4–6	2/18 (11.1%)	2/32 (6.3%)	
Preoperative severe arterial damage	7 (31.8%)	6 (10.3%)	0.037∗
Follow-up period (months)	8.5 ± 7.6 (2–36)	7.7 ± 6.0 (2–29)	0.68
≥3 months	19 (86.4%)	49 (84.5%)	1.00
≥6 months	11 (50%)	31 (53.4%)	0.81
Interval between image and TACE (months)^†^			0.084
<1 month	26/29 (89.7%)	62/87 (71.3%)	
1-2 months	1/29 (3.4%)	18/87 (20.7%)	
2-3 months	2/29 (6.9%)	7/87 (8.0%)	

Age is presented as median (range).

AFP, DCP, nodule size, and follow-up period are presented as mean ± standard deviation.

Range of follow-up period is shown in the parentheses.

*P < 0.05.

^†^Interval between preoperative image and treatment was evaluated on a per treatment session basis.

HCV: hepatitis C virus; HBV: hepatitis B virus; AFP: alpha-fetoprotein; DCP: des-gamma-carboxyprothrombin; BCLC: Barcelona Clinic Liver Cancer; PS: performance status (Eastern Cooperative Oncology Group classification); TACE: transcatheter arterial chemoembolization; EPI: epirubicin; CDDP: cisplatin; MPT: miriplatin.

**Table 2:** Results of logistic regression analysis.

Factors	Odds ratio (95% CI)	*P* value
Sex (female)	0.39 (0.12–1.17)	0.10
HBV infection	0.30 (0.046–1.49)	0.16
Tumor size (mm^2^)	1.00 (1.00-1.00)	0.13
AFP (ng/mL)	1.00 (1.00-1.00)	0.16
DCP (mAU/mL)	1.00 (1.00-1.00)	0.14
BCLC stage C	0.77 (0.24–2.43)	0.66
BCLC stage D	36.55 (0.70–3635.77)	0.078
History of TACE	0.78 (0.27–2.24)	0.64
Warming miriplatin	12.35 (2.90–90.0)	0.0028∗∗
Miriplatin dose (mg)	0.99 (0.96–1.01)	0.43
Severe hepatic arterial damage	0.59 (0.10–2.73)	0.52

**P < 0.01.

CI: confidence interval; HBV: hepatitis B virus; AFP: alpha-fetoprotein; DCP: des-gamma-carboxyprothrombin; BCLC: Barcelona Clinic Liver Cancer; TACE: transcatheter arterial chemoembolization.

**Table 3:** Evaluation of the effect of anticancer agents on the hepatic artery.

	Epirubicin/cisplatin (*n* = 43)	Nonwarmed miriplatin (*n* = 30)	Warmed miriplatin (*n* = 46)
Damage grade			
0 (no damage)	25 (58.1%)	22 (73.3%)	44 (95.7%)
1 (irregularity)	5 (11.6%)	6 (20.0%)	2 (4.3%)
2 (narrowing)	1 (2.4%)	0 (0%)	0 (0%)
3 (stenosis)	3 (7.0%)	0 (0%)	0 (0%)
4 (occlusion)	9 (20.9%)	2 (6.7%)	0 (0%)
Damage level			
1 (PHA)	1/18 (5.6%)	0/8 (0%)	0/2 (0%)
2 (lobar branch)	3/18 (16.7%)	0/8 (0%)	0/2 (0%)
3 (segmental branch)	6/18 (33.3%)	3/8 (37.5%)	1/2 (50%)
4 (subsegmental branch)	8/18 (44.4%)	5/8 (62.5%)	1/2 (50%)
A-P shunt formation			
Yes	2 (4.7%)	3 (10.0%)	0 (0%)
No	41 (95.3%)	27 (90.0%)	46 (100%)
Number of sessions			
1	28 (65.1%)	24 (80.0%)	27 (58.7%)
2	10 (23.3%)	5 (16.7%)	14 (30.4%)
3	1 (2.3%)	1 (3.3%)	4 (8.7%)
4–6	4 (9.3%)	0 (0%)	1 (2.2%)

Data in this table were obtained from consensus of two radiologists.

PHA: proper hepatic artery; A-P shunt: arterioportal shunt.

**Table 4:** Adverse events.

	Nonwarmed group (29 sessions)	Warmed group (87 sessions)	*P* value
AST (IU/L), *n* (%)	46.8 ± 22.4	59.1 ± 50.0	0.14
Grade 1	10 (34.5%)	24 (27.6%)	
Grade 2	5 (17.2%)	20 (23.0%)	
Grade 3	3 (10.3%)	26 (29.9%)	
Grades 4-5	0 (0)	0 (0)	0.0083∗∗
ALT (IU/L), *n* (%)	35.9 ± 15.6	50.6 ± 56.2	0.32
Grade 1	8 (27.6%)	27 (31.0%)	
Grade 2	1 (3.4%)	14 (16.1%)	
Grade 3	4 (13.8%)	22 (25.3%)	
Grades 4-5	0 (0)	0 (0)	0.0068∗∗
T-Bil (mg/dL), *n* (%)	1.03 ± 0.75	0.81 ± 0.39	0.25
Grade 1	5 (17.2%)	29 (33.3%)	
Grade 2	3 (10.3%)	8 (9.2%)	
Grade 3	0 (0)	0 (0)	
Grades 4-5	0 (0)	0 (0)	0.22
WBC (/*µ*L), *n* (%)	3666 ± 1477	3840 ± 1604	0.59
Grade 1	1 (3.4%)	7 (8.0%)	
Grade 2	1 (3.4%)	7 (8.0%)	
Grade 3	3 (10.3%)	9 (10.3%)	
Grades 4-5	0 (0)	0 (0)	0.38
Hb (g/dL), *n* (%)	12.0 ± 2.0	11.6 ± 1.9	0.32
Grade 1	3 (10.3%)	19 (21.8%)	
Grade 2	1 (3.4%)	10 (11.5%)	
Grade 3	1 (3.4%)	3 (3.4%)	
Grades 4-5	0 (0)	0 (0)	0.060
Plt (×10^4^/*µ*L), *n* (%)	9.2 ± 4.5	10.8 ± 5.0	0.20
Grade 1	5 (17.2%)	26 (29.9%)	
Grade 2	7 (24.1%)	19 (21.8%)	
Grade 3	2 (6.9%)	14 (16.1%)	
Grades 4-5	0 (0)	0 (0)	0.11
Eosinophilia^†^	13/27 (48.1%)	22/62 (35.5%)	0.26
Pyrexia, *n* (%)			
Grade 1	11 (37.9%)	35 (40.2%)	
Grade 2	0 (0)	7 (8.0%)	
Grades 3–5	0 (0)	0 (0)	0.22
Vomiting, *n* (%)			
Grade 1	1 (3.4%)	3 (3.4%)	
Grade 2	1 (3.4%)	0 (0)	
Grades 3–5	0 (0)	0 (0)	0.42
Liver infarction	0	0	—
Liver abscess	0	0	—
Bile duct necrosis	0	0	—

Preoperative values of AST, ALT, T-Bil, WBC, Hb, and Plt are presented as mean ± standard deviation in the first row.

*P* values in the first row are for comparison of preoperative values, while values in the bottom row are for comparison of CTCAE grades.

**P < 0.01.

^†^Data on eosinophilia were missing in 2 sessions with the nonwarmed group and in 25 sessions with the warmed group.

AST: aspartate transaminase; ALT: alanine transaminase; T-Bil: total bilirubin; WBC: white blood cell; Hb: hemoglobin; Plt: platelets.
